# Barriers to accessibility of medicines for hyperlipidemia in low- and middle-income countries

**DOI:** 10.1371/journal.pgph.0002905

**Published:** 2024-02-12

**Authors:** Chaoyang Li, Garrison Spencer, Muhammad Jami Husain, Rachel Nugent, Deon Auzenne, Deliana Kostova, Patricia Richter

**Affiliations:** 1 Division of Global Health Protection, Global Health Center, Centers for Disease Control and Prevention, Atlanta, Georgia, United States of America; 2 Center for Global Noncommunicable Diseases, RTI International, Research Triangle Park, North Carolina, United States of America; 3 Department of Psychology, Howard University, Washington, District of Columbia, United States of America; PLOS: Public Library of Science, UNITED STATES

## Abstract

Despite the high burden of hyperlipidemia and the effectiveness of treatment, evidence suggests that the accessibility of hyperlipidemia medicines can be low in many low- and middle-income countries (LMICs). The aim of this study was to identify common barriers to the accessibility of medicines for hyperlipidemia in LMICs. A multimethod analysis and multiple data sources were used to assess the accessibility and barriers of medicines for hyperlipidemia in selected LMICs. The overall median availability of statins for hyperlipidemia in public facilities was 0% and 5.4%, for originators and generics, respectively. In private facilities, median availability was 13.3% and 35.9%, for originators and generics, respectively. Statin availability was lowest in Africa and South-East Asia. Private facilities generally had higher availability than public facilities. Statins are less affordable in lower-income countries, costing around 6 days’ wages per month. Originator statins are less affordable than generics in countries of all income-levels. The median cost for statin medications per month ranges from a low of $1 in Kenya to a high of $62 in Mexico, with most countries having a median monthly cost between $3.6 and $17.0. The key informant interviews suggested that accessibility to hyperlipidemia medicines in LMICs faces barriers in multiple dimensions of health systems. The availability and affordability of statins are generally low in LMICs. Several steps could be implemented to improve the accessibility of hyperlipidemia medicines, including private sector engagement, physician education, investment in technology, and enhancement of health systems.

## Introduction

Cardiovascular diseases (CVD) are the leading cause of death worldwide, and 80% of CVD deaths occur in low- and middle-income countries (LMICs) [1]. Hyperlipidemia (or dyslipidemia) is one of the important risk factors for CVD [2]. Hyperlipidemia encompasses disorders of lipoproteins, including elevations of total cholesterol, low-density lipoprotein cholesterol (LDL-C), non-high-density lipoprotein cholesterol (non-HDL-C), and triglycerides, as well as low HDL cholesterol.

Based on the global estimates from the Noncommunicable Disease Risk Factor Collaboration (NCD-RisC), age-standardized mean non-HDL cholesterol concentration increased in LMICs, particularly in East and South-East Asia and sub-Saharan Africa and Melanesia, whereas it decreased substantially in high-income western regions and central and eastern Europe from 1980 to 2018 [3]. Data from the Global Burden of Disease (GBD) Study 2019 suggest that a total of 3.78 million deaths from ischemic heart disease (IHD) worldwide could be attributable to high LDL-C concentrations, accounting for 44.3% of IHD deaths. A total of 0.61 million deaths from ischemic stroke could be attributable to high LDL-C levels, accounting for 22.4% of ischemic stroke deaths [4]. The age-standardized death rates for IHD and ischemic stroke attributable to high LDL-C levels have reduced by about 35% from 1990 to 2019 globally. These rates have decreased in most high-income countries, however, the rates did not change in many Asian and Africa countries or even significantly increased in Central Asia and East Asia.

Statins, inhibitors of HMG-CoA reductase, and the rate-limiting enzyme of cholesterol biosynthesis, have been clinically used as first line agents to reduce LDL-C [5]. Statins are the only lipid-modifying medication on the World Health Organization (WHO) Model List of Essential Medicines. Statins are the leading recommended treatment globally and make up most lipid-modifying medicines currently in use [6,7]. Compared to the high-income countries [8–10], the prevalence of statin uses among eligible people for the treatment of dyslipidemia or the secondary prevention of cardiovascular diseases in the LMICs [11,12] remains low.

The WHO defines access as “having medicines continuously available and affordable at public or private health facilities or medicine outlets that are within one hour’s walk from the homes of the population” [13]. Despite the high burden of hyperlipidemia and the effectiveness of treatment, evidence suggests that the accessibility of hyperlipidemia medicines can be extremely low in many LMIC contexts [14]. In LMICs, several characteristics could impede access to statins, including high prices, lack of coverage in health benefit packages, and low availability in pharmacies or other healthcare facilities. The aim of this study was to identify common barriers to the accessibility of medicines for hyperlipidemia in LMICs. By reviewing international databases and peer-reviewed literature as well as conducting key informant interviews, this study describes conditions in a range of LMICs and the status of access to medicines for hyperlipidemia by region and income levels.

## Methods

In this study, we used a multimethod approach to review, collect and analyze data from various sources including international databases, literature review of published articles, and key informant interviews.

### International database sources

Three data sources that were reviewed and analyzed included Health Action International (HAI), Service Availability and Readiness Assessment (SARA), and Management Sciences for Health (MSH).

#### Health Action International (HAI)

The HAI Database of Medicine Prices, Availability, Affordability, and Price Components was created in collaboration with WHO in 2001 in order to develop a reliable methodology for collecting and analyzing medicine prices, availability, and affordability across different sectors and regions and to publish those data in a publicly available website to improve transparency and advocate for appropriate national policies [15]. The database contains availability, price, and affordability data for both generic and originator drugs, as well as for both private and public medicine outlets (i.e., pharmacy, clinic, or other health facility). Availability of individual medicines is defined as “the percentage of medicine outlets in which the medicine was found on the day of data collection”. Simvastatin is one of 14 drugs included in the HAI/WHO global list of medicines survey template, though therapeutically equivalent medicines may be added if they are widely used, and other types of statins are reported in the database.

The HAI database reports the median unit price of medicines, which represents the final dispensed price paid for a medicine, whether it be purchased by a patient, government, or insurance provider. This price includes any value-added tax, goods, and services tax, or dispensing fees but does not include any drug registration fees, patient fees for service, co-payments, informal charges, or discounts and rebates. Affordability is defined as the number of days’ wages of the lowest-paid unskilled national government employee required to purchase a month’s supply of statin therapy (30 tablets). According to HAI standards, medicines are usually considered affordable if they cost one day’s wages or less for an entire course of treatment for an acute condition or for a 30-day supply for a chronic condition.

#### Service Availability and Readiness Assessment (SARA)

The SARA is a health facility survey tool created by WHO to assess and monitor the service availability and readiness of the health sector and to create evidence to assist in the planning and management of health systems. The SARA methodology includes surveying at least 150 public and private facilities and is designed to generate a set of tracer indicators of health system service availability and readiness, including the availability of 14 medicines from the WHO Essential Medicines List (EML) [16].

#### Management Sciences for Health (MSH)

The International Medical Products Price Guide has been published by the MSH since 1986 and in collaboration with WHO since 2000 and is meant to contribute to equitable access to health products and essential medicines by making price information more widely available in order to improve procurement for the lowest possible prices [17]. The guide includes prices from suppliers (including international aid organizations and procurement agencies that maintain a warehouse and supply items directly to customers) and buyers (usually government agencies).

The three international data sources (i.e., HAI data, SARA data, and MSH guide) are publicly accessible. Country income level in 2017 was based on World Bank data [18]. Health expenditure as a percent of GDP and health expenditure per capita data were obtained from the WHO Global Health Expenditure Database [19]. Disease burden (all cause mortality and DALYs) attributed to high LDL-C were obtained from the Global Burden of Disease Study 2017 [20].

### The clinical practice and experience of countries from literature reviews

To identify peer-reviewed literature assessing the accessibility of hyperlipidemia medicines in LMIC contexts, a literature search of the PubMed database was conducted in January 2020 and updated in June 2022. The search was limited to publications in English, and that had been published from January 1, 2010, through May 31, 2022. A second search was conducted with several countries from Latin America, Africa, South Asia, and East Asia that were selected for their large populations and the likelihood of having data available. Google and Google Scholar searches were conducted to identify grey literature and online databases containing information on the availability, accessibility, or affordability of hyperlipidemia medicines. A snowball technique of searching the reference lists of retrieved included studies was used to identify additional relevant studies. The PubMed search strategy and terms are available in **S1** and **S2 Tables**. The Endnote 21 reference management tool (https://endnote.com/?language=en) and Covidence systematic review management tool (https://www.covidence.org/) were used for the title and abstract screening, full text review, and data extraction of included studies. Each study was assessed independently by two researchers.

### Key informant interviews

To supplement limited data availability and inform barriers to access in LMIC contexts, interviews were conducted with key stakeholders, including representatives from the pharmaceutical industry, nongovernmental organizations (NGOs), multilateral organizations, and academia, in February and March 2020. The interview was designed by RTI, and the initial list of experts invited for interviews was developed collaboratively by RTI, CDC, and CDC Foundation. In addition, experts from the Ministries of Health or other relevant government agencies from five LMICs were also invited for their specialized knowledge of accessibility issues for hyperlipidemia medicines.

The interviewees were asked a series of standard questions on factors that impact demand for lipid-modifying medicines from the patient perspective, features of the market that act as barriers to access, systematic features of LMIC markets that contribute to limited accessibility, ways to mitigate barriers to accessibility, and about initiatives that have been successful in improving access to lipid-modifying medicines.

### Data summarization and analysis

Microsoft 365 Excel (https://www.microsoft.com/en-us/microsoft-365) was used to summarize, merge, and analyze data. Median availability and mean affordability of statins were calculated by WHO region and WB income levels.

## Results

### Availability of medicines for hyperlipidemia

The HAI database contains availability data for any statin across 46 countries representing each WHO region and income level (**Table 1**). The overall median availability of statins for hyperlipidemia among included countries was 0% and 5.4% for originators and generics in public facilities, and 13.3% and 35.9% in private facilities, respectively.

**Table 1:** Median availability and mean affordability of statins by region and income levels, HAI database, 2001–2015.

Characteristic	Availability, %, median					Affordability, number of days’ wage*, mean				
		Public facility		Private facility			Public facility		Private facility	
	Num of Countries	Originator	Generic	Originator	Generic	Num of Countries	Originator	Generic	Originator	Generic
**Overall**	46	0.0%	5.4%	13.3%	35.9%	26	6.1	2.9	6.7	2.8
**WHO Region**										
Africa	10	0.0%	0.0%	3.4%	11.1%	3	--†	--	11.4	4.9
Americas	10	0.0%	3.3%	20.0%	58.6%	6	--	--	11.2	3.4
East Mediterranean	11	0.0%	45.5%	30.2%	86.1%	7	--	1.4	4.1	1.4
Europe	6	17.1%	75.2%	35.7%	88.6%	5	4.8	4.0	5.8	5.3
South-East Asia	3	0.0%	0.0%	1.6%	20.0%	2	--	--	1.6	0.7
Western Pacific	6	13.4%	10.6%	10.0%	33.3%	3	7.0	3.2	6.0	2.5
**WB Income Level**										
Low	9	0.0%	0.0%	0.0%	15.2%	5	--	5.8	--	3.8
Lower middle	17	0.0%	0.0%	0.0%	22.2%	10	5.7	5.4	6.6	6.0
Upper middle	16	0.0%	11.1%	33.3%	68.8%	9	6.2	2.0	6.4	2.0
High	4	0.0%	47.3%	57.2%	80.9%	2	--	--	5.0	1.5

Note

* Affordability is defined as the number of days wages of the lowest-paid unskilled national government employee required to purchase a month supply of statin therapy (30 tablets).

†Data unavailable.

WHO = World Health Organization; WB = World Bank.

Statin availability was lowest in Africa and South-East Asia, with no availability of statins in public facilities (**Table 1**). Private facilities generally had higher availability. The lowest availability was in originator statins in public facilities, except for in Western Pacific, where the higher availability of originator statins in public facilities may be driven by China.

There appeared to be a clear and positive relationship between country income level and availability (**Table 1**). Additionally, generic statins were more available than originator statins in both public and private facilities in countries of all income levels. The highest level of availability was found for generic statins in private facilities, with this gap being more pronounced in upper-middle-income countries. HAI data show greater availability of statins in higher-income countries. The detailed availability data by country from the HAI database can be found in **S3 Table**.

Of the 15 countries with SARA reports available on the WHO website, 10 reported the overall level of availability of statins in healthcare facilities. These countries are all in sub-Saharan Africa dated from 2010 to 2016 and ranged from 0% in Burkina Faso to 5% in Sierra Leone and Zambia [21] (**Table 2)**. In the MSH guide, the lowest price was reported by a buyer for 20 mg lovastatin at less than half a cent per pill, while the most expensive price was reported by a buyer for 40 mg simvastatin for $0.20 (**Table 3**) [17]. At least one type or up to seven types of statins were listed in the EML among 11 out of 14 countries identified (**Table 4**). The most common types of statins were atorvastatin and simvastatin, followed by fluvastatin, pravastatin, and rosuvastatin. Other types of statins included lovastatin, ezetimibe, and ezetimibe-simvastatin combination.

**Table 2:** Statin availability in public and private health facilities, SARA reports, 2010–2016.

Country	WB income classification	Statin availability (%)	Year of SARA report
Burkina Faso	Low	0%	2014
Dem. Rep. of Congo	Low	2%	2014
Togo	Low	2%	2012
Benin	Low	3%	2015
Mauritania	Lower middle	3%	2016
Niger	Low	3%	2015
Tanzania	Low	3%	2012
Uganda	Low	3%	2013
Sierra Leone	Low	5%	2012
Zambia	Lower middle	5%	2010

Note: SARA = Service availability and readiness assessment; WB = World Bank.

**Table 3:** MSH international medical products price guide for prices (US $) on lipid-lowering agents, 2015.

			Supplier			Buyer	
Medicine	Defined daily dose	Number of prices	High/low price ratio	Median price, US $	Number of prices	High/low price ratio	Median price, US $
Atorvastatin 10 mg	20 mg	2	3.0	0.05	4	2.2	0.02
Atorvastatin 20 mg	20 mg	3	8.0	0.10	3	2.9	0.05
Atorvastatin 40 mg	20 mg	NA	--	--	2	3.8	0.07
Gemfibrozil 600 mg	20 mg	NA	--	--	3	2.2	0.04
Lovastatin 20 mg	45 mg	NA	--	--	1	--	0.01
Simvastatin 10 mg	30 mg	NA	--	--	1	--	0.01
Simvastatin 20 mg	30 mg	2	1.5	0.02	1	--	0.05
Simvastatin 40 mg	30 mg	NA	--	--	1	--	0.20

**Table 4:** Accessibility to medicines of hyperlipidemia and country profiles, 2017.

Region	Country (Ref #)	World Bank income level	Health expenditure, % of GDP	Health expenditure, per capita US dollars	Death attributable to high LDL (% of total death)	DALYs attributable to high LDL (% of total DALYs)	Guideline for hyperlipidemia management	Essential Medicines List (EML)	Availability of statins in clinical practice	Median cost of statins, per month or 30-day supply, or per unit	Barriers to obtaining hyperlipidemia medicines
***Africa***											
	Cameroon [14]	Lower middle	3.5%	$49.8	2147 (1.0%)	61015 (0.4%)	NA	NA	• Atorvastatin available in 20% of private community outlets, 5.9% of urban outlets, and 4% of semi-urban outlets • Simvastatin available in 50% of private community outlets, 17.6% of urban outlets, and 8% of semi-urban outlets.	• Atorvastatin cost $13.9 for a 30-day supply, equal to 6.9 days wage • Simvastatin cost $26.9 for a 30-day supply, equal to 13.4 days’ wage	• Statin medications were largely purchased by out-of-pocket payments due to the absence of universal health coverage and few health insurance schemes
	Ghana [15]	Lower middle	3.4%	$68.0	7972 (3.8%)	212379 (1.7%)	NA	• Atorvastatin • Fluvastatin • Rosuvastatin • Simvastatin	• Statins were found in none of the nine community-based health planning services surveyed (2013)	NA	NA
	Kenya [16–18,38]	Lower middle	4.1%	$65.1	4473 (1.5%)	129537 (0.7%)	• Strongly recommends statin treatment for those with a CVD risk greater than 20% or with known CVD • Recommends statin treatment be considered for those with moderate risk (between 10% and 20%)	• Atorvastatin	• Simvastatin was available in only 10, or 21% of the facilities	• $1 per treatment per month	• Cost represented a major barrier to long term adherence • County-level variation in medicine procurement and distribution • Private facility outlets were not included in the program • Contradictions between treatment guidelines and the EML
	Nigeria [19,20]	Lower middle	3.8%	$73.9	28527 (1.7%)	732007 (0.6%)	NA	NA	NA	• Generics $8.90 per month • Originator brands $17.00 per month	• Under-dosing
	South Africa [21,36,39]	Upper middle	8.7%	$534.4	15653 (2.8%)	374022 (1.3%)	• Recommends statin treatment be considered for patients with low and moderate CVD risk • Recommends immediately initiated for high-risk patients with high cholesterol	• Atorvastatin • Pravastatin • Rosuvastatin • Simvastatin	• Overall, 93% of facilities had a statin in stock	• $3.6 per month (58 South African Rand; 9.7% of a household’s capacity-to-pay)	• Long wait times deterring patients from returning for cholesterol monitoring
***Eastern Mediterranean***											
	Iran [22]	Lower middle	8.4%	$481.6	49741 (13.0%)	1141211 (5.8%)	NA	• Atorvastatin • Rosuvastatin • Simvastatin • Lovastatin	NA	NA	NA
***South-East Asia***											
	Bangladesh [23,36]	Lower middle	2.4%	$37.4	44847 (5.4%)	1211031 (2.8%)	NA	• Simvastatin	◾ Consultations are provided at low cost and medicines are free of charge to patients. ◾ Many patients purchasing medicines out-of-pocket.	• $5.2 per month (450 Bangladeshi Taka, BDT, 15.8% of a household’s capacity-to-pay)	
	India [24,36,40]	Lower middle	2.9%	$57.6	592129 (6.5%)	16914661 (3.6%)	• Recommends statins as the first line of treatment for hyperlipidemia • Add fibrates if targeted levels are not met on the maximum tolerated dose of statins	• Atorvastatin • Simvastatin		• $3.8 per month (296 Indian Rupee, INR, 16.6% of a household’s capacity-to-pay	• Low per capita prescribing rates
	Indonesia [25,41]	Lower middle	2.9%	$111.4	107383 (6.4%)	3151277 (4.0%)	• Recommends statin treatment for type 2 diabetes patients who are over 40 years of age or at high CVD risk	• Atorvastatin • Simvastatin	NA	NA	NA
***Americas***											
	Brazil [26,36,42]	Upper middle	9.5%	$940.4	95510 (7.0%)	2291836 (3.5%)	• Recommends statin treatment as the first line therapy • Add ezetimibe when statin therapy alone is unable to achieve LDL cholesterol goals	• Atorvastatin • Fluvastatin • Lovastatin • Pravastatin • Simvastatin	• 68.1% of statin users reported accessing their medication at an SUS pharmacy free of charge, 16.7% from a private pharmacy, 13.8% from pharmacy with a co-payment, and 1.5 percent from other sources.	• $14.2 per month (73 Brazilian Real, BRL, 3.9% of a household’s capacity-to-pay)	• Poor adherence
	Mexico [28]	Upper middle	5.5%	$506.9	50027 (7.1%)	1109947 (3.3%)	NA	• Atorvastatin • Fluvastatin • Pravastatin • Rosuvastatin • Simvastatin • Ezetimibe • Ezetimibe-simvastatin combination	• 23.0% of patients diagnosed with dyslipidemia have initiated treatment.	• $62.0 per month (792 Mexican Pesos)	• Variability between healthcare systems • Failure to calculate low and high cardiovascular risk, patients with hyperlipidemia were treated in the same way • Length of treatment was inadequate • High-intensity statin therapy was unavailable
***Western Pacific***											
	China [29,36,43]	Upper middle	5.1%	$437.3	872065 (8.5%)	19255113 (5.1%)	• Recommends medium-intensity statins for initial treatment, then adjusting dose according to individual efficacy and tolerance, adding ezetimibe if necessary	• Atorvastatin • Lovastatin • Simvastatin	NA	• $3.7 per month (25 Chinese Yuan, RMB, 2.4% of household capacity-to-pay)	• Low utilization of generics • Tendency of prescribing originators
	Philippines [30]	Lower middle	4.0%	$123.7	49747 (7.8%)	1445544 (4.4%)	NA	• Atorvastatin • Pravastatin • Simvastatin	• Atorvastatin was available in 38.9% of public facilities and 83.3% of private facilities • Pravastatin was available in 0% of public facilities and 23.8% of private facilities • Rosuvastatin was available in 27.8% of public facilities and 64.3% of private facilities • Simvastatin was available in 61.1% of public facilities and 81% of private facilities	• Atorvastatin in private facilities: median unit price: $0.68; in public facility median unit price: $0.19 • Pravastatin in private facility median unit price: $0.46 • Rosuvastatin in private facilities: median unit price: $1.53; in in public facility median unit price: $ 0.36 • Simvastatin in private facilities: median unit price: $0.60; in public facility median unit price: $0.08	• Low statin medication availability in the public facility • Patients purchased their medicines from private pharmacies • Increasing the patient’s out-of-pocket spending on drugs
	Vietnam [31–34]	Lower middle	4.7%	$140.1	38445 (6.2%)	876615 (3.4%)	NA	NA	• None of the five rural CHCs surveyed had a statin available in 2010 • Only one of the five urban CHCs had a statin available Simvastatin or lovastatin was available in seven of 15 primary care facilities (46.7%)	NA	• Public health insurance did not cover statins at CHCs • Patients must travel to district level hospitals in order to have their insurance cover a statin prescription

**Fig 1:**
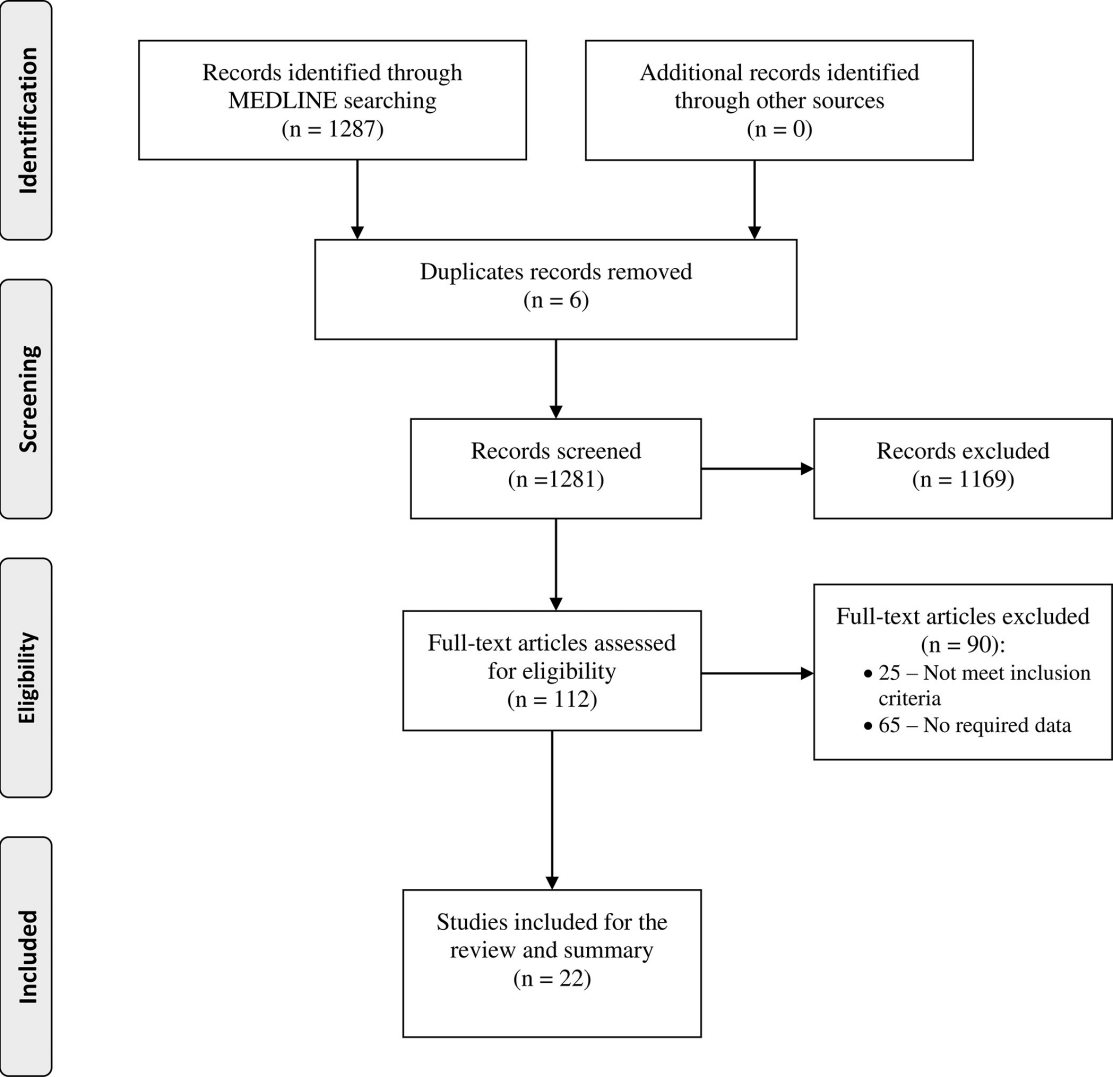
PRISMA flow diagram.

The literature search assessing the accessibility of hyperlipidemia medicines in LMIC contexts yielded 1,287 articles, of which 112 were selected for full-text review, and 22 studies that contained accessibility data were included in the review (**Fig 1**). Among included studies, two large multi-country epidemiological studies, the Africa Middle East Cardiovascular Epidemiological (ACE) Study [22] and the Prospective Urban Rural Epidemiological (PURE) Study [23], were reviewed.

Data from the literature review showed the limited availability of statins in clinical practice in LMICs. In Cameroon, Atorvastatin and simvastatin were available in 50% of private community outlets and were more available at urban outlets than semi-urban outlets [24]. A survey conducted in Ghana in 2013 showed that statins were unavailable at all nine Community-based Health Planning Services (CHPS, lowest level of care in Ghana) or the nine health centers surveyed [25]. Statins were available in one of the three district hospitals surveyed and in both of the two regional hospitals surveyed. In Kenya, qualitative research among providers at the Kenyatta National Hospital in Nairobi indicated that statins were available and, that stockouts were rare, but that cost represents a major barrier to long term adherence [26]. A survey conducted in Nyandarua County, Kenya, in 2018 found that simvastatin was available in only 10 out of 47 primary healthcare facilities, or 21% [27]. In Delta State of Nigeria, 150 community pharmacists were surveyed about statin use. While the majority of pharmacists correctly identified statins as the first line treatment for high cholesterol, less than half were able to identify the most common side effect of statin therapy [28]. A review of prescriptions in patients admitted at a tertiary hospital in southwestern Nigeria between January 2012 and August 2013 found that 59 patients (out of 1,280) received a total of 62 statin prescriptions–two patients were prescribed more than one statin–and only 29.0% of these statins were prescribed by their generic name [29]. In South Africa, although the overall percentage of facilities that had at least one of atorvastatin, simvastatin, or rosuvastatin in stock on the day of the survey was high (93.0%), qualitative research by the investigators revealed that there was widespread belief in the community that stockouts were common [30].

Bangladesh has a publicly funded healthcare system in which consultations were provided at low cost and medicines were free of charge to patients, however, about half of the physicians employed in the public district and local hospitals were not satisfied with the availability of medicines at their facilities, leading to many patients purchasing medicines out-of-pocket [31]. In India, analysis of prescription data collected by Intercontinental Medical Statistics (IMS) Health found that, as of January 2010, there were 259 unique statin products available from 65 manufacturers, with atorvastatin accounting for 81.1% of the products and 84.8% of statin sales [32]. In Indonesia, only 1.47% were on statin treatment among those at high CVD risk (2.01% in urban areas, 0.91% in semi-urban areas, and 0.94% in rural areas) [33].

In the Philippines, statins were more available at private facilities (ranging from 23.8% to 83.3%) than at public facilities (ranging from 0.0% to 61.1%) [34]. In Vietnam, a survey done in Phunhuan district between 2009 and 2011 found that simvastatin or lovastatin was available in seven of 15 primary care facilities [35]. A survey conducted in 2010 in Dong Hy district found that statins were not available at any of the 18 CHCs included in the study [36]. However, a survey of Commune Health Center (CHC) capacity conducted in 2017 in Hai Dong province found that none of the five rural CHCs had a statin available, and only one of the five urban CHCs had a statin available [37]. Although statins may be available at some CHCs, public health insurance did not cover them at this level of care, so patients must travel to district-level hospitals in order to have their insurance cover a statin prescription [38].

In Brazil, 68.1% of statin users reported accessing their medication at an Sistema Único de Saúde (SUS, Brazil’s publicly funded healthcare system) pharmacy free of charge, 16.7% from a private pharmacy, 13.8% from the pharmacy in the Popular Pharmacy Program with a co-payment, and 1.5% from other sources [39]. A survey conducted between July 2014 and May 2015 of patients aged 18 years and older from 1,305 primary healthcare centers in 272 cities found that the prevalence of statin use was 9.3%. In Mexico, the CARMELA study, published in 2010, showed that the prevalence of dyslipidemia in Mexico City was 50.1% among adults aged 25 to 64 years but that only 21.7% of those who were prescribed a lipid-modifying medication were currently taking that medication [40].

### Affordability and price of medicines for hyperlipidemia

Where available, originator statins required at least twice as many days’ wages as the lowest cost generic statins in Africa, the Americas, Eastern Mediterranean, South-East Asia, and Western Pacific, while there is a smaller affordability gap in Europe. (**Table 1**). The median cost for statin medications per month ranged from a low of $1 in Kenya to a high of $62 in Mexico. The majority of countries had a median monthly cost between $3.6 and $17.0. On average, statins appeared to be unaffordable by region using the HAI standard. Statins were less affordable in lower-income countries, with both originators and generics costing around 6 days’ wages on average (**Table 1**). The full set of price and affordability data from the HAI database can be found in **S4 Table**.

The cost of statin treatment accounted for less than 10% of a household’s capacity-to-pay in China (2.4%), Brazil (3.9%), and South Africa (9.7%) [23]. In contrast, this cost accounts for greater than 15% of a household’s capacity-to-pay in Bangladesh (15.8%) and India (16.6%) (**Table 4**). Originator statins were less affordable than generics in countries at any income-level.

### Guidelines for hyperlipidemia management

Based on the literature review, six out of 14 countries had guidelines and recommendations on statin treatment (**Table 4**). The Kenya National Guidelines for Cardiovascular Diseases Management strongly recommends statin treatment for those with a CVD risk greater than 20% or with known CVD, and recommends statin treatment be considered for those with moderate risk (between 10% and 20%) who do not reach treatment goals with lifestyle management [41]. The South African dyslipidemia guideline consensus statement recommends statin therapy be considered for patients with low and moderate cardiovascular risk when lifestyle interventions do not control high LDL cholesterol and be immediately initiated for high-risk patients with high cholesterol [42]. Indian guidelines recommend statins as the first line of treatment for hyperlipidemia and recommend adding fibrates if targeted levels are not met on the maximum tolerated dose of statins [43]. Indonesia’s treatment guidelines for type 2 diabetes indicate statin treatment for those over 40 years of age or at high CVD risk [44]. Brazil’s Guidelines on Cardiovascular Prevention recommend statin treatment as the first line of therapy and recommends the addition of ezetimibe when statin therapy alone is unable to achieve LDL cholesterol goals [45]. China’s hyperlipidemia treatment guidelines recommend medium-intensity statins for initial treatment, then adjusted according to individual efficacy and tolerance, adding ezetimibe if necessary [46].

### Barriers to the accessibility of lipid-modifying medications in clinical practice

Kenya was the first country to receive assistance through the Novartis Access program, which made available for purchase a portfolio of NCD medicines, including simvastatin, for $1 per treatment per month by the Mission for Essential Drugs and Supplies (MEDS)–a main distributor to Kenyan public and non-profit health facilities [47]. A cluster-randomized controlled trial of the program that included 127 health facilities in eight counties showed that simvastatin was found at three or fewer facilities at follow-up and was not available even at well-stocked hospital pharmacies. In this study, barriers to the availability of simvastatin were identified, including county-level variation in medicine procurement and distribution; $1 per treatment per month still being above international median reference prices; many patients purchasing medicines at private sector outlets which were not included in the program; and, contradictions between treatment guidelines and the EML. In Nigeria, under-dosing (prescribed daily dose compared to the defined daily dose) was observed in 56.0% of the statin prescriptions [29].

A baseline assessment of health service capacity that was conducted in South Africa in 2015 to identify gaps in the continuum of care for cardiovascular diseases at 86 health facilities in two districts identifies several barriers to obtaining hyperlipidemia medications. Only approximately half of health facilities had total cholesterol or LDL cholesterol tests, resulting in patients needing to be referred to higher-level facilities for diagnosis [30]. An additional barrier identified by both patients and providers was long wait times deterring patients from returning for cholesterol monitoring. In India, per capita, prescribing rates among patients remain 20 times lower than those in the United States and Canada [32].

In China, despite the reduced price, there continued to be a low utilization of generics and a tendency of prescribing originators. For example, in one of the hospital systems, prescribing generic simvastatin–the lowest cost statin–accounted for 0.1% of statin prescriptions despite being between 68.0% and 77.0% cheaper than the most frequently prescribed statin, originator atorvastatin (30.7% of prescriptions), to which it was therapeutically similar [48]. In Vietnam, public health insurance did not cover statins at the commune health centers, so patients must travel to district-level hospitals in order to have their insurance cover a statin prescription [38].

In Brazil, poor adherence was self-reported by 6.5% of statin users, with the most common cause being a personal decision not to take medicine (30.7%), forgetting to take medicine (24.1%), lack of access (23.5%), adverse events (10.6%), and other causes (11.2%) [39]. A review of hyperlipidemia treatment in Mexico identified several barriers to better outcomes, including the low proportion of the population who have ever had a lipid screen, variability between healthcare systems (with many using random capillary cholesterol measurements instead of the recommended fasting lipid profile), and a failure to calculate the cardiovascular risk that results in low- and high-risk hyperlipidemia cases being treated in the same way [49]. Additionally, the length of treatment is inadequate, and high-intensity statin therapy is unavailable in most public healthcare systems.

The main barriers by domains and by the components of accessibility [14] as identified in the key informant interviews are summarized in **Table 5**. Statins did not seem to be prioritized in existing initiatives to improve access to hyperlipidemia medicines. No interviewee was able to provide an example of an existing initiative that had a quantifiable impact specifically on the accessibility of medicines for hyperlipidemia. Accessibility to hyperlipidemia medicines in LMICs faced barriers in multiple dimensions of health systems, among providers, and among patients. Though the barriers to accessibility provided by the interviewees were numerous and complex, and statins had not been prioritized in past accessibility initiatives, there were ideas on how to improve the situation moving forward.

**Table 5:** Key barriers to accessibility of statins identified in key informant interviews.

Domain	Accessibility component	Barrier
**Leadership and governance**	Accessibility	• Lipid-lowering drugs are not authorized at low levels of care or can only be prescribed by specialists.
	Affordability	• Newly formed national or supranational regulatory agencies in LMICs may require dossiers for well-established drugs that increase the cost and make economies of scale more difficult to accomplish.
**Essential medicines**	-	• Corruption during the medicine procurement process.
	Accessibility	• In many LMIC context, physicians prescribe statins at low doses to avoid myalgia though this is not best practice.
	Accessibility	• Procurement patterns in LMICs often means that the medicines that are available change frequently. This results in medicines of different sizes, colors, doses, and side effects, which can affect adherence.
	Accessibility	• Issues with procurement and transportation can cause questions about medicine reliability, due to medicines arriving without the correct packaging or technical instructions, not having been stored properly or for too long a time, or they may be counterfeit in the first place.
	Affordability	• In remote settings, pharmacists may mark up prices knowing that the patient does not have the opportunity to compare prices and will not go elsewhere.
	Affordability	• Trend of in-house physicians at pharmacies may cause conflicts of interest.
	Affordability	• Lipid-lowering drugs often only available in private pharmacies, which are often more expensive.
	-	• Research suggests that presence on a country’s EML has an effect on availability. Both national EMLs and treatment guidelines are often quite outdated, and research suggests that presence on a country’s EML has an effect on availability.
**Health financing**	-	• Lipid profile tests needed to diagnose hyperlipidemia are often prohibitively expensive.
	Affordability	• Lipid-lowering drugs are unaffordable.
	Affordability	• Factors such as being reimbursable, covered by the public facility, or part of insurance schemes affect accessibility. If lipid-lowering medicines are covered but the financing system is dysfunctional, that will affect accessibility as well.
**Health information systems**	-	• There is very poor data coming out of LMICs on NCDs and hyperlipidemia medicine distribution and lipid levels are not being input into District Health Information Software 2 (DHIS2). While most lipid-lowering drugs utilized in LMICs are statins, which are mostly generic, unlike the US and Europe, it is difficult to obtain market authorization information about these drugs for LMICs. Therefore, in LMIC contexts it is hard to know who is making statins, where they are selling them, and what type of quality they are.
	-	• There is a lack of information on how frequently statins go into shortage. There does not seem to be evidence of a lack of access from a manufacturing standpoint.
**Human resources**	-	• Lack of awareness among physicians that testing for lipid levels should be a part of a package of services for those at risk of CVD, even at high-level facilities.
	-	• Little training in medical schools about how to communicate CVD risk to patients
	-	• Lack of lower-level healthcare workers capable of preforming the necessary lab work to diagnose hyperlipidemia.
	-	• Competition between different healthcare professions can inhibit task sharing that might allow different cadres of health workers to diagnose or treat hyperlipidemia.
	-	• Lack of specialized physicians; patients can only see general practitioners who do not have specialized knowledge for treating hyperlipidemia
**Service delivery**	Accessibility	• Hard for patients to receive adequate diagnostics, prescription, monitoring, and follow-up due to poorly functioning health system.
	Accessibility	• The distance to the pharmacy is prohibitive.
	Accessibility	• In remote settings, the only option for distribution is sometimes private facilities which are difficult and more expensive to access for multinational companies, in part due to limited infrastructure.
	Availability	• Lipid-lowering drugs are not available due to stockouts or other supply chain issues.
**Patient**	-	• Low demand for hyperlipidemia medicine from patients (hyperlipidemia is asymptomatic)
	-	• Side effects of hyperlipidemia medicines
	-	• Poor understanding of cardiovascular risk, do not comprehend 10-year CVD risk profiles.
	-	• Hyperlipidemia does not have as strong of an association with stroke as hypertension, which incentivizes patients to adhere to hypertension treatment
	-	• Psychological aspect of having to take a drug for the rest of one’s life.
	-	• Difficult to monitor the progression of treatment due to requiring blood draws.
	Affordability	• Intrahousehold competition for resources

When asked about ways to overcome these barriers and improve accessibility to medicines for hyperlipidemia, the most frequent response related to the role of technology and digital health applications. These tools could be used in many ways, including identifying patients at high risk through social media using artificial intelligence and thereby reducing the need for screening; diagnostics and monitoring hyperlipidemia through wearable devices; training or supplementing community health workers with eLearning apps; dispensing medicines through mobile pharmacies; and providing hyperlipidemia information and referrals in local languages. Concerns about digital technology use include the limited scope of many apps, patient privacy, difficulty with regulation, and problems with uptake among older patients. New lipid-modifying drugs or delivery methods, such as a drug that is injected twice a year, could improve access and adherence. Also, strengthening the voice of patients through advocacy groups that exist for other types of diseases could also increase demand. An especially interesting proposal was to integrate solutions whereby the pharmaceutical company would not only provide the medicine, but also support the diagnostic and treatment management processes. An integrated provider could potentially relieve some of the burden of seeking care from the patient. This could possibly be approached through the tendering process.

**Fig 2:**
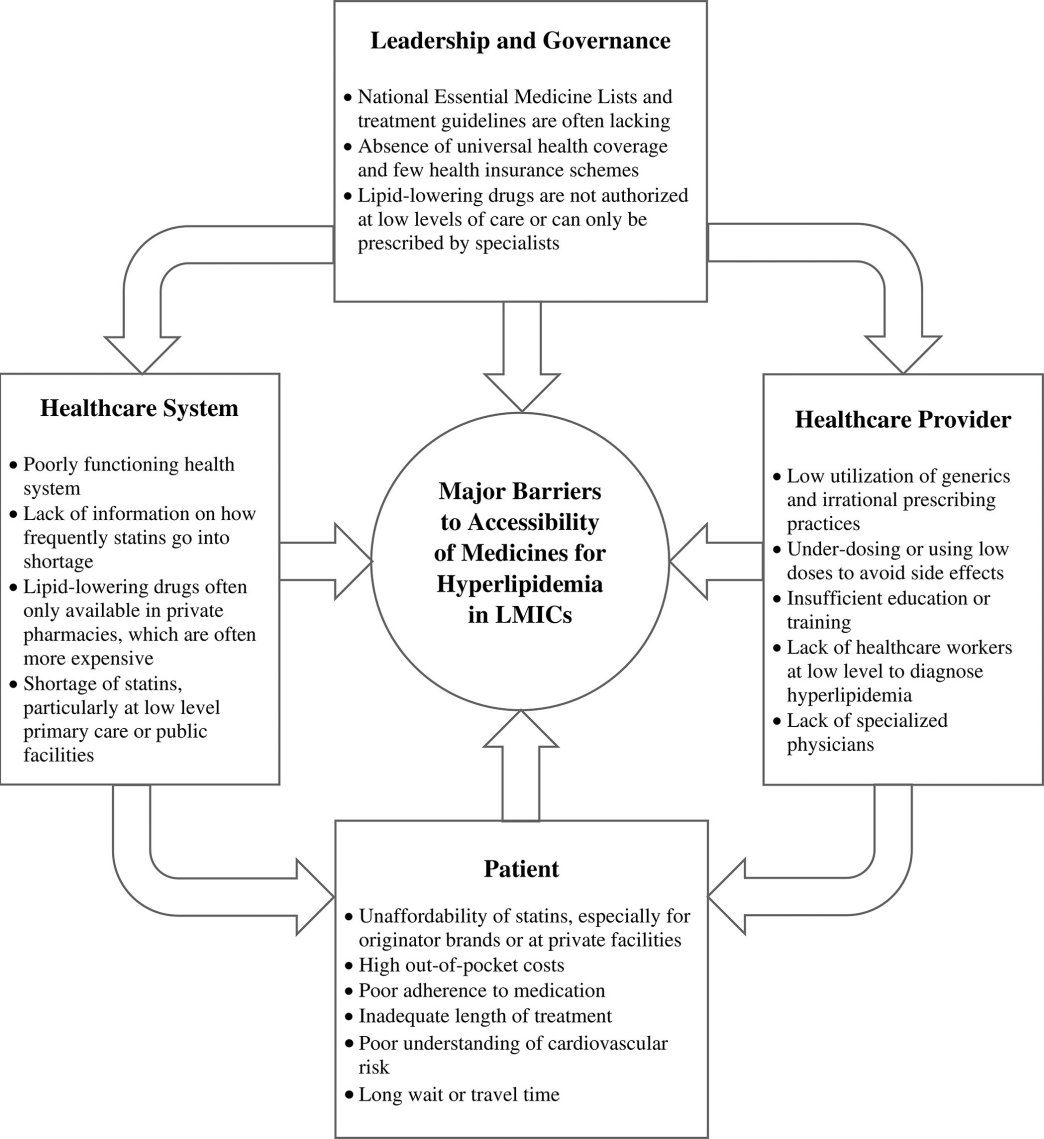
Major barriers to accessibility of medicines for hyperlipidemia in LMICs.

By combining the results from multiple data sources, the major barriers to accessibility of medicines for hyperlipidemia in LMICs was summarized in **Fig 2.**

## Discussion

This study examined the barriers to the accessibility of medicines for hyperlipidemia in LMICs. We found some evidence of the poor accessibility of statins in selected countries based on our literature review and cross-country analysis. The availability of medicines for hyperlipidemia based on the international databases including HAI data, SARA data, and MSH guide was under 15% for originators and under 40% for generics and was predominant in private facilities. The lowest statin availability was identified in Africa and South-East Asia. The most common types of statins were atorvastatin and simvastatin, followed by fluvastatin, pravastatin, and rosuvastatin.

There was limited availability of statins in clinical practice in LMICs. In Africa, statins were unavailable at most community-based health or clinical centers and cost represents a major barrier to long term adherence of statin use. In South-East Asia, statins were more available at private facilities than at public facilities. Statins were either unavailable or not covered by public health insurance at community health centers so that the patients traveled to higher level health facilities to obtain the medicines. Although statins were free of charge to patients in a publicly funded healthcare system in some countries, however, the availability of statins at the health facilities was unsatisfactory which resulted in many patients purchasing the medicines out-of-pocket. In Americas, statins were also free of charge in publicly funded healthcare system, however, the utilization of satins remained low.

High costs of originator satins were identified as a major barrier where generic statins were unavailable. Originator statins at least doubled the price of generic statins in Africa, the Americas, Eastern Mediterranean, South-East Asia, and Western Pacific. Statins appeared to be unaffordable according to the HAI standard. The costs of statin treatment were even higher in South-East Asia than Africa, Americas, and Western Pacific.

The main strength of this study included the use of a multimethod approach and multiple data sources from international databases, a literature review of published country-specific data, and key informant interviews to assess the barriers to accessibility of medicines for hyperlipidemia in LMICs. However, there were several limitations in this study. First, data provided by countries that have conducted a survey using HAI methodology were not verified by HAI/WHO. The database provides periodic results from various countries with available data in different years. Second, some of the data were collected in 2001, and the conditions in those countries may have changed substantially. Third, the number of countries reporting statin availability and affordability in the HAI database was small, and the reporting time varied from 2001 to 2015. The comparability of data across countries, regions, and income levels should be interpreted with caution. Fourth, the number of countries included in the literature review was small due to a lack of studies identified. Therefore, the generalizability of these findings to all other LMICs could be limited. Future research is needed to assess the relationships between the availability and affordability of statins as well as the impact of the accessibility of the medicines on cardiovascular disease burden (e.g., mortality, DALY, or quality of life) in LMICs.

### Conclusions

The findings of this study suggest that the accessibility of medicines for hyperlipidemia was limited and barriers to the accessibility might exist at various levels of the health systems in LMICs. The overall availability and affordability were low. Statins were more available in private facilities than public facilities, and more available in urban areas than in rural areas. Generic statins were more available than originator statins in both public and private facilities. Statins were unaffordable to patients regardless of region and income level.

### Recommendations

To tackle the barriers identified in this study, there are several steps that could be implemented to improve the accessibility of hyperlipidemia medicines in LMICs.

### First, private sector engagement could be prioritized

Pharmaceutical companies are actively engaged with access programs and play an important role in LMIC contexts; however, statins do not seem to be prioritized in these programs. Exploring how to emphasize the importance of hyperlipidemia treatment to these companies and innovative ways of engaging them, such as integrated diagnosis and treatment, could improve the prominence of hyperlipidemia medicines in these programs and increase accessibility.

### Second, physician education and training could be emphasized

Hyperlipidemia is an asymptomatic chronic condition, and there is little demand for treatment by patients. Therefore, patients rely on physicians to advocate for them and educate them on the risks of hyperlipidemia. Unfortunately, even in many high-level facilities in LMICs, lipid profiles are not being ordered, indicating a need for greater awareness of the impact of managing lipids in cardiovascular disease prevention. Also, prescribing practices should be reviewed, and guidelines provided so that patients are encouraged to avail themselves of quality generic medicines.

### Third, investment in technology could be encouraged

LMICs have opportunities to adopt modern technology systems without going through intermediary steps. For example, using wearable devices to diagnose and monitor hyperlipidemia, mobile apps to encourage adherence or train community health workers, or new technologies to distribute medicines represents some of the possibilities for technology to improve access to hyperlipidemia medicines.

### Fourth, enhancement of health systems could be implemented

Many of the barriers identified in the key informant interviews stem from weak health systems. While not specific to hyperlipidemia medicines, investments in health financing, governance, information systems, service delivery, and the health workforce will certainly combine to improve NCD care and accessibility to medicines, including for hyperlipidemia.

## Supporting information

S1 Table PubMed search results for accessibility, affordability, and availability of medicines for hyperlipidemia in LMIC contexts. (DOCX)

S2 Table PubMed search results for accessibility, affordability, and availability of medicines for hyperlipidemia in specific countries. (DOCX)

S3 Table Availability (%) of statins from Health Action International (HAI) database. (DOCX)

S4 Table Price and affordability of statins from Health Action International (HAI) database. (DOCX)
