# Supplementary material for: Barriers to accessibility of medicines for hyperlipidemia in low- and middle-income countries
Source: PLOS Glob Public Health. 2024 Feb 12;4(2):e0002905. doi: 10.1371/journal.pgph.0002905 (PMC10861044; doi:10.1371/journal.pgph.0002905)
Supplement: S1 Table — (DOCX) [file pgph.0002905.s001.docx]

**S1 Table**: PubMed search results for accessibility, affordability, and availability of medicines for hyperlipidemia in LMIC contexts

| Search | Query | Original literature search ("2010/01/01"[Date - Publication] : "2020/01/07"[Date - Publication])) AND "English"[Language]) | Update literature search ("2020/01/08"[Date - Publication] : "2022/05/31"[Date - Publication])) AND "English"[Language]) |
| --- | --- | --- | --- |
| #1 | Search (LMIC OR LLMIC OR LMICS OR LLMICS OR “low-income countr*” OR “low- and middle-income countr*” OR “middle-income countr*” OR “developing countr*” OR “under developed countr*” OR “underdeveloped countr*” OR “low income countr*” OR “middle income countr*” OR “third world countr*” OR “poor countr*” OR “developing countries”[MeSH Terms]) Sort by: Best Match | 141335 | 25929 |
| #2 | Search ((availab* AND medicine*) OR (availab* AND medication*) OR (availab* AND drug*) OR (availab* AND treatment*) OR (affordab* AND medicine*) OR (affordab* AND medication*) OR (affordab* AND drug*) OR (affordab* AND treatment*) OR (access* AND medicine*) OR (access* AND medication*) OR (access* AND drug*) OR (access* AND treatment*) OR (quality AND medicine*) OR (quality AND medication*) OR (quality AND drug*) OR (quality AND treatment*) OR (shortage* AND medicine*) OR (shortage* AND medication*) OR (shortage* AND drug*) OR (shortage* AND treatment*) OR (barrier* AND medicine*) OR (barrier* AND medication*) OR (barrier* AND drug*) OR (barrier* AND treatment*) OR (price* AND medicine*) OR (price* AND medication*) OR (price* AND drug*) OR (price* AND treatment*) OR (acceptab* AND medicine*) OR (acceptab* AND medication*) OR (acceptab* AND drug*) OR (acceptab* AND treatment*) OR (adherence AND medicine*) OR (adherence AND medication*) OR (adherence AND drug*) OR (adherence AND treatment*) OR affordability OR acceptability OR accessibility OR accessible OR affordable OR acceptable OR quality) Sort by: Best Match | 2898913 | 675801 |
| #3 | Search (cholesterol[MeSH Terms] OR dyslipidemia[MeSH Terms] OR hyperlipidemia OR hypercholesterolemia OR lipids OR cholesterol OR dyslipidemia) Sort by: Best Match | 1502025 | 149391 |
| #4 | #1 AND #2 AND #3 Sort by: Best Match | 432 | 157 |
