# Supplementary material for: Barriers to accessibility of medicines for hyperlipidemia in low- and middle-income countries
Source: PLOS Glob Public Health. 2024 Feb 12;4(2):e0002905. doi: 10.1371/journal.pgph.0002905 (PMC10861044; doi:10.1371/journal.pgph.0002905)
Supplement: S2 Table — (DOCX) [file pgph.0002905.s002.docx]

**S2 Table**: PubMed search results for accessibility, affordability, and availability of medicines for hyperlipidemia in specific countries

| Search | Query | Original Literature Search ("2000/01/01"[Date - Publication] : "2020/01/07"[Date - Publication])) AND "English"[Language]) AND "humans"[Filter] | Update  Literature Search ("2020/01/08"[Date - Publication] : "2022/05/31"[Date - Publication])) AND "English"[Language]) AND "humans"[Filter] |
| --- | --- | --- | --- |
| #1 | Search (cholesterol[MeSH Terms] OR dyslipidemia[MeSH Terms] OR hyperlipidemia OR hypercholesterolemia) Sort by: [pubsolr12] | 233228 | 16546 |
| #2 | Search (availab* OR affordab* OR access*) Sort by: [pubsolr12] | 1813452 | 375659 |
| #3 | Search ("Hydroxymethylglutaryl-CoA Reductase Inhibitors"[Mesh] OR "Fibric Acids"[Mesh] OR "Ezetimibe"[Mesh] OR "Cholestyramine Resin"[Mesh] OR "bile acid sequestrant" OR "Proprotein Convertase 9"[Mesh] OR "Niacin"[Mesh] OR atorvastatin OR fluvastatin OR lovastatin OR pravastatin OR rosuvastatin OR simvastatin OR pitavastatin OR cerivastatin OR mevastatin OR statin OR statins OR medication OR medications OR medicine OR medicines OR prescription OR prescriptions OR drug OR drugs) Sort by: [pubsolr12] | 14964720 | 2138651 |
| #4 | Search ("south africa" OR kenya OR ghana OR nigeria OR mexico OR brazil OR peru OR india OR bangladesh OR china OR vietnam OR indonesia) Sort by: [pubsolr12] | 3035207 | 1030106 |
| #5 | #1 AND #2 AND #3 AND #4 Sort by: [pubsolr12] | 453 | 245 |
