# Supplementary material for: Barriers to accessibility of medicines for hyperlipidemia in low- and middle-income countries
Source: PLOS Glob Public Health. 2024 Feb 12;4(2):e0002905. doi: 10.1371/journal.pgph.0002905 (PMC10861044; doi:10.1371/journal.pgph.0002905)
Supplement: S3 Table — (DOCX) [file pgph.0002905.s003.docx]

**S3 Table**: Availability (%) of Statins from Health Action International (HAI) Database

| **Country** | **Subregion** | **WHO Region** | **WB Income Classification** | **Medicine and Dose** | **Availability in Public Sector (%)** | | **Availability in Private Sector (%)** | | **Data Year** | **Mean/**  **Median** |
| --- | --- | --- | --- | --- | --- | --- | --- | --- | --- | --- |
|  |  |  |  |  | **Originator** | **Generic** | **Originator** | **Generic** |  |  |
| Afghanistan |  | EMR | Low | Atorvastatin 20 mg | 0.0% | 4.5% | 4.0% | 24.0% | 2011 | Mean |
|  |  |  |  | Simvastatin 20 mg | 0.0% | 45.5% | 6.0% | 94.0% | 2011 | Mean |
| Bolivia |  | Americas | LM | Atorvastatin 10 mg | 0.0% | 7.2% | 0.0% | 70.0% | 2008 | Mean |
|  |  |  |  | Simvastatin 20 mg | 0.0% | 0.0% | 0.0% | 6.7% | 2008 | Mean |
| Brazil | Rio Grande do Sul | Americas | UM | Atorvastatin 10 mg | 0.0% | NA | 53.3% | NA | 2008 | Mean |
|  |  |  |  | Simvastatin 20 mg | 0.0% | 0.0% | 33.3% | 70.0% | 2008 | Mean |
| Burkina Faso |  | Africa | Low | Simvastatin 20 mg | 0.0% | 0.0% | 14.7% | 11.8% | 2009 | Mean |
| Burundi |  | Africa | Low | Simvastatin 20 mg | NA | 0.0% | NA | 0.0% | 2013 | Mean |
| China | Shaanxi Province | WPR | UM | Atorvastatin 20 mg | 69.4% | 11.1% | 65.3% | 20.8% | 2014 | Mean |
|  | Shaanxi Province |  |  | Lovastatin 20 mg | 0.0% | 11.1% | 1.4% | 36.1% | 2014 | Mean |
|  | Shaanxi Province |  |  | Simvastatin 20 mg | 20.6% | 7.4% | 44.4% | 36.1% | 2014 | Mean |
|  | Shandong Province |  |  | Atorvastatin 10 mg | 10.0% | 20.0% | 10.0% | 0.0% | 2004 | Median |
|  | Shanghai |  |  | Lovastatin 20 mg | 0.0% | 13.3% | 0.0% | 10.0% | 2006 | Median |
|  | Shandong Province |  |  | Lovastatin 20 mg | 0.0% | 50.0% | 0.0% | 45.0% | 2004 | Median |
|  | Shanghai |  |  | Simvastatin 20 mg | 40.0% | 36.7% | 55.0% | 0.0% | 2006 | Median |
|  | Shandong Province |  |  | Simvastatin 20 mg | 25.0% | 5.0% | 25.0% | 0.0% | 2004 | Median |
| Colombia |  | Americas | UM | Atorvastatin 10 mg | 3.3% | 4.3% | 30.5% | 76.3% | 2008 | Mean |
|  |  |  |  | Lovastatin 20 mg | 0.0% | 100.0% | 1.7% | 93.2% | 2008 | Mean |
|  |  |  |  | Simvastatin 20 mg | 3.3% | 6.7% | 18.6% | 35.6% | 2008 | Mean |
| Ecuador |  | Americas | UM | Atorvastatin 10 mg | 0.0% | 10.0% | 73.3% | 80.0% | 2008 | Mean |
|  |  |  |  | Simvastatin 20 mg | 0.0% | 3.3% | 36.7% | 70.0% | 2008 | Mean |
| El Salvador |  | Americas | LM | Lovastatin 20 mg | 0.0% | 5.8% | 5.8% | 30.8% | 2006 | Median |
| Ethiopia |  | Africa | Low | Lovastatin 20 mg | 0.0% | 0.0% | 0.0% | 12.0% | 2004 | Median |
| Fiji |  | Western Pacific | UM | Lovastatin 20 mg | NA | NA | 5.6% | 86.1% | 2004 | Median |
| Ghana |  | Africa | LM | Lovastatin 20 mg | 0.0% | 0.0% | 0.0% | 0.0% | 2004 | Median |
| Haiti |  | Americas | Low | Atorvastatin 10 mg | 0.0% | 0.0% | 8.6% | 14.3% | 2011 | Mean |
|  |  | Americas | Low | Simvastatin 20 mg | 0.0% | 1.9% | 0.0% | 57.1% | 2011 | Mean |
| India | Delhi | SEAR | LM | Atorvastatin 10 mg | 0.0% | 15.7% | 12.5% | 97.5% | 2011 | Mean |
|  | Delhi |  |  | Simvastatin 20 mg | 0.0% | 1.2% | 42.5% | 40.0% | 2011 | Mean |
|  | Chennai |  |  | Lovastatin 20 mg | 0.0% | 0.0% | 7.5% | 55.0% | 2004 | Median |
|  | Haryana |  |  | Lovastatin 20 mg | 0.0% | 0.0% | 0.0% | 13.3% | 2004 | Median |
|  | Mararashtra 12 districts |  |  | Lovastatin 20 mg | 0.0% | 0.0% | 0.0% | 16.7% | 2004 | Median |
|  | Karnataka |  |  | Lovastatin 20 mg | 0.0% | 0.0% | 0.0% | 12.5% | 2004 | Median |
|  | Maharashtra 4 regions |  |  | Lovastatin 20 mg | 0.0% | 10.5% | 0.0% | 10.4% | 2005 | Median |
|  | Rajasthan |  |  | Lovastatin 20 mg | 0.0% | 0.0% | 0.0% | 20.0% | 2003 | Median |
| Indonesia |  | SEAR | LM | Atorvastatin 10 mg | 30.0% | 0.0% | 53.1% | 0.0% | 2010 | Mean |
|  |  |  |  | Simvastatin 20 mg | 0.0% | 54.8% | 1.6% | 57.8% | 2010 | Mean |
|  |  |  |  | Lovastatin 20 mg | 0.0% | 6.7% | 0.0% | 20.7% | 2004 | Median |
| Iran |  | EMR | UM | Atorvastatin 20 mg | 16.7% | 90.0% | 36.7% | 96.7% | 2014 | Mean |
|  |  |  |  | Simvastatin 20 mg | NA | 60.0% | NA | 93.3% | 2014 | Mean |
| Jordan |  | EMR | UM | Simvastatin 20 mg | 5.6% | 0.0% | 55.0% | 85.0% | 2004 | Median |
| Kazakhstan |  | Europe | UM | Lovastatin 20 mg | 0.0% | 0.0% | 5.0% | 0.0% | 2004 | Median |
| [Kenya](javascript:hwopen('notes.php?Srv_ID=124%27)) |  | Africa | LM | Lovastatin 20 mg | 0.0% | 0.0% | 0.0% | 10.3% | 2004 | Median |
| Kyrgyzstan |  | Europe | LM | Simvastatin 20 mg | NA | NA | 0.0% | 20.0% | 2015 | Mean |
| Lao PDR |  | WPR | LM | Atorvastatin 20 mg | 16.7% | 10.0% | 38.9% | 33.3% | 2013 | Mean |
| Lebanon |  | EMR | UM | Atorvastatin 10 mg | 0.0% | 13.3% | 93.3% | 96.7% | 2013 | Mean |
|  |  |  |  | Simvastatin 20 mg | 0.0% | 96.7% | 96.7% | 100.0% | 2013 | Mean |
| Malaysia |  | WPR | UM | Lovastatin 20 mg | 0.0% | 100.0% | 3.1% | 84.4% | 2004 | Median |
|  |  |  |  | Simvastatin 20 mg | 90.0% | 0.0% | 81.3% | 68.8% | 2004 | Median |
| Mauritius |  | Africa | UM | Atorvastatin 10 mg | 0.0% | 93.3% | 6.7% | 100.0% | 2008 | Mean |
|  |  |  |  | Simvastatin 20 mg | 0.0% | 0.0% | 13.3% | 23.3% | 2008 | Mean |
| Mexico | Mexico City | Americas | UM | Atorvastatin 10 mg | 0.0% | 0.0% | 33.3% | 0.0% | 2009 | Mean |
|  |  |  |  | Pravastatin 30 mg | 0.0% | 61.5% | 20.0% | 60.0% | 2009 | Mean |
|  |  |  |  | Simvastatin 20 mg | 0.0% | 0.0% | 0.0% | 6.7% | 2009 | Mean |
| Moldova |  | Europe | LM | Simvastatin 20 mg | NA | 46.0% | NA | 50.0% | 2011 | Mean |
| Mongolia |  | WPR | LM | Atorvastatin 20 mg | NA | 0.0% | NA | 20.0% | 2012 | Mean |
|  |  |  |  | Simvastatin 20 mg | NA | 6.5% | NA | 68.6% | 2012 | Mean |
| Nicaragua |  | Americas | LM | Atorvastatin 10 mg | 0.0% | 0.0% | 45.2% | 32.3% | 2008 | Mean |
|  |  |  |  | Simvastatin 20 mg | 3.3% | 0.0% | 35.5% | 83.9% | 2008 | Mean |
| Oman |  | EMR | High | Atorvastatin 20 mg | 0.0% | 0.0% | 31.2% | 0.0% | 2007 | Mean |
|  |  |  |  | Simvastatin 20 mg | NA | NA | 18.8% | 75.0% | 2007 | Mean |
| Pakistan |  | EMR | LM | Lovastatin 20 mg | 0.0% | 0.0% | 29.2% | 12.5% | 2004 | Median |
| Peru |  | Americas | UM | Lovastatin 20 mg | 0.0% | 3.8% | 8.3% | 35.4% | 2005 | Median |
| Philippines |  | WPR | LM | Lovastatin 20 mg | 0.0% | 0.0% | 0.0% | 2.0% | 2005 | Median |
| Russia | Tatarstan | Europe | UM | Atorvastatin 10 mg | 80.0% | 100.0% | 90.0% | 100.0% | 2011 | Mean |
|  |  |  |  | Simvastatin 20 mg | 53.3% | 100.0% | 56.7% | 100.0% | 2011 | Mean |
|  |  |  |  | Simvastatin 40 mg | 6.7% | 73.3% | 50.0% | 96.7% | 2011 | Mean |
| Sao Tome & Principe |  | Africa | LM | Simvastatin 20 mg | 0.0% | 0.0% | 0.0% | 22.2% | 2008 | Mean |
| Saudi Arabia |  | EMR | High | Atorvastatin 10 mg | 0.0% | 5.7% | 93.3% | 86.7% | 2015 | Mean |
|  |  |  |  | Simvastatin 10 mg | 0.0% | 94.3% | 73.3% | 90.0% | 2015 | Mean |
| South Africa | Kwazulu natal State | Africa | UM | Simvastatin 10 mg | NA | NA | 95.0% | 0.0% | 2001 | Median |
| Sudan |  | EMR | LM | Atorvastatin 20 mg | 0.0% | 87.5% | 0.0% | 86.1% | 2013 | Mean |
|  |  |  |  | Simvastatin 20 mg | 0.0% | 62.5% | 0.0% | 50.0% | 2013 | Mean |
| Syria |  | EMR | Low | Lovastatin 20 mg | NA | NA | 0.0% | 96.5% | 2003 | Median |
| Tajikistan |  | Europe | Low | Simvastatin 20 mg | 3.4% | 13.8% | 0.0% | 16.1% | 2013 | Mean |
| Tanzania |  | Africa | Low | Simvastatin 20 mg | NA | 0.0% | NA | 16.7% | 2012 | Mean |
|  |  |  | LM | Lovastatin 20 mg | 0.0% | 0.0% | 0.0% | 4.2% | 2004 | Median |
| Thailand |  | SEAR | UM | Atorvastatin 10 mg | 20.0% | 0.0% | 85.7% | 0.0% | 2006 | Median |
|  |  |  |  | Simvastatin 10 mg | 0.0% | 85.0% | 33.3% | 100.0% | 2006 | Median |
| Tunisia |  | Africa | LM | Simvastatin 20 mg | 0.0% | 0.0% | 95.1% | 0.0% | 2004 | Median |
| Ukraine |  | Europe | LM | Atorvastatin 20 mg | 17.1% | 82.9% | 40.0% | 91.4% | 2012 | Mean |
|  |  |  |  | Simvastatin 20 mg | 25.7% | 77.1% | 31.4% | 88.6% | 2012 | Mean |
| United Arab Emirates |  | EMR | High | Simvastatin 20 mg | 22.2% | 88.9% | 100.0% | 73.9% | 2006 | Median |
| USA | Boston area | Americas | High | Simvastatin 20 mg | NA | NA | 41.0% | 94.1% | 2015 | Mean |
| Yemen |  | EMR | Low | Lovastatin 20 mg | 0.0% | 0.0% | 0.0% | 0.0% | 2006 | Median |

Abbreviations used in the table: EMR – Eastern Mediterranean Region, SEAR – South-East Asia Region, WPR – Western Pacific Region, LM – lower middle-income, UM – upper middle-income, NA – not available.

***Table S4****:* **Price and affordability of statins from Health Action International (HAI) Database**

| Country | Subregion | WHO Region | WB Income Classification | Year  of Survey | Drug and Dose | Local Currency Unit  (LCU) |  | Daily Wage, LCU | Originator or Lowest Price Generic | Public Sector | | Private Sector | |
| --- | --- | --- | --- | --- | --- | --- | --- | --- | --- | --- | --- | --- | --- |
|  |  |  |  |  |  |  |  |  |  | Median Treatment Price per month, LCU | No. of Days’ wage | Median Treatment Price per month, LCU | No. of Days’ wage |
| Afghanistan |  | EM | Low | 2011 | Atorvastatin 20mg | Afghani |  | 150 | LPG |  |  | 109.2 | 0.7 |
|  |  |  |  | 2011 | Simvastatin 20mg | Afghani |  | 150 | LPG | 120.0 | 0.8 | 160.0 | 1.1 |
| Brazil | Rio Grande do Sul | Americas | UM | 2008 | Simvastatin 20mg | Reals |  | 12.73 | LPG |  |  | 47.7 | 3.8 |
|  |  |  |  | 2008 | Atorvastatin 10mg | Reals |  | 12.73 | ORIG |  |  | 104.9 | 8.2 |
|  |  |  |  | 2008 | Simvastatin 20mg | Reals |  | 12.73 | ORIG |  |  | 106.4 | 8.4 |
| Burkina Faso |  | Africa | Low | 2009 | Simvastatin 20mg | FCFA |  | 1023 | LPG |  |  | 9920.0 | 9.7 |
|  |  |  |  | 2009 | Simvastatin 20mg | FCFA |  | 1023 | ORIG |  |  | 21412.5 | 20.9 |
| China | Shaanxi Province | WP | UM | 2012 | Atorvastatin 20mg | Yuan Renminbi |  | 33.33 | LPG | 223.5 | 6.7 | 53.5 | 1.6 |
|  | Shaanxi Province |  |  | 2014 | Atorvastatin 20mg | Yuan Renminbi |  | 37.33 | LPG | 203.5 | 5.5 | 192.8 | 5.2 |
|  | Shaanxi Province |  |  | 2012 | Simvastatin 20mg | Yuan Renminbi |  | 33.33 | LPG | 58.9 | 1.8 | 56.2 | 1.7 |
|  | Shaanxi Province |  |  | 2014 | Simvastatin 20mg | Yuan Renminbi |  | 37.33 | LPG | 32.2 | 0.9 | 47.1 | 1.3 |
|  | Shaanxi Province |  |  | 2010 | Simvastatin 20mg | Yuan Renminbi |  | 25.33 | LPG | 55.5 | 2.2 | 49.2 | 1.9 |
|  | Shaanxi Province |  |  | 2012 | Atorvastatin 20mg | Yuan Renminbi |  | 33.33 | ORIG | 315.4 | 9.5 | 255.0 | 7.7 |
|  | Shaanxi Province |  |  | 2014 | Atorvastatin 20mg | Yuan Renminbi |  | 37.33 | ORIG | 309.0 | 8.3 | 252.0 | 6.8 |
|  | Shaanxi Province |  |  | 2010 | Atorvastatin 20mg | Yuan Renminbi |  | 25.33 | ORIG | 315.4 | 12.5 | 278.5 | 11.0 |
|  | Shaanxi Province |  |  | 2012 | Simvastatin 20mg | Yuan Renminbi |  | 33.33 | ORIG | 107.1 | 3.2 | 102.8 | 3.1 |
|  | Shaanxi Province |  |  | 2014 | Simvastatin 20mg | Yuan Renminbi |  | 37.33 | ORIG | 103.6 | 2.8 | 98.1 | 2.6 |
|  | Shaanxi Province |  |  | 2010 | Simvastatin 20mg | Yuan Renminbi |  | 25.33 | ORIG | 138.0 | 5.4 | 113.5 | 4.5 |
| Colombia |  | Americas | UM | 2008 | Lovastatin 20mg | Colombian Pesos |  | 15383 | LPG |  |  | 9000.0 | 0.6 |
|  |  |  |  | 2008 | Simvastatin 20mg | Colombian Pesos |  | 15383 | LPG |  |  | 56100.0 | 3.6 |
|  |  |  |  | 2008 | Simvastatin 20mg | Colombian Pesos |  | 15383 | ORIG |  |  | 232800.0 | 15.1 |
| Ecuador |  | Americas | UM | 2008 | Atorvastatin 10mg | USD |  | 6.67 | LPG |  |  | 21.1 | 3.2 |
|  |  |  |  | 2008 | Simvastatin 20mg | USD |  | 6.67 | LPG |  |  | 20.7 | 3.1 |
|  |  |  |  | 2008 | Atorvastatin 10mg | USD |  | 6.67 | ORIG |  |  | 44.9 | 6.7 |
|  |  |  |  | 2008 | Simvastatin 20mg | USD |  | 6.67 | ORIG |  |  | 53.8 | 8.1 |
| Egypt |  | EM | LM | 2013 | Atorvastatin 10mg | Egyptian Pounds |  | 40 | LPG |  |  | 68.5 | 1.7 |
|  |  |  |  | 2013 | Simvastatin 20mg | Egyptian Pounds |  | 40 | LPG |  |  | 85.7 | 2.1 |
|  |  |  |  | 2013 | Atorvastatin 10mg | Egyptian Pounds |  | 40 | ORIG |  |  | 128.5 | 3.2 |
|  |  |  |  | 2013 | Simvastatin 20mg | Egyptian Pounds |  | 40 | ORIG |  |  | 171.4 | 4.3 |
| Haiti |  | Americas | Low | 2011 | Atorvastatin 10mg | Gourdes |  | 200 | LPG |  |  | 525.0 | 2.6 |
|  |  |  |  | 2011 | Simvastatin 20mg | Gourdes |  | 200 | LPG |  |  | 425.0 | 2.1 |
| India | NCT Delhi | SEA | LM | 2011 | Atorvastatin 10mg | Indian Rupees |  | 247 | LPG |  |  | 252.0 | 1.0 |
|  |  |  |  | 2011 | Simvastatin 20mg | Indian Rupees |  | 247 | LPG |  |  | 145.2 | 0.6 |
|  |  |  |  | 2011 | Atorvastatin 10mg | Indian Rupees |  | 247 | ORIG |  |  | 252.0 | 1.0 |
|  |  |  |  | 2011 | Simvastatin 20mg | Indian Rupees |  | 247 | ORIG |  |  | 540.0 | 2.2 |
| Indonesia |  | SEA | LM | 2010 | Simvastatin 20mg | Indonesian Rupiah |  | 36500 | LPG | 20253.9 | 6.0 | 21000.0 | 0.6 |
| Iran |  | EM | UM | 2007 | Atorvastatin 20mg | Iranian Rials |  | 108400 | LPG | 33000.0 | 0.3 | 33000.0 | 0.3 |
|  |  |  |  | 2007 | Simvastatin 20mg | Iranian Rials |  | 108400 | LPG | 30000.0 | 0.3 | 30000.0 | 0.3 |
|  |  |  |  | 2014 | Simvastatin 20mg | Iranian Rials |  | 270000 | LPG | 51000.0 | 0.2 | 51000.0 | 0.2 |
|  |  |  |  | 2007 | Atorvastatin 20mg | Iranian Rials |  | 108400 | ORIG |  |  | 912000.0 | 8.4 |
| Kyrgyzstan |  | Europe | LM | 2010 | Simvastatin 20mg | Som |  | 26.67 | LPG |  |  | 687.2 | 25.8 |
|  |  | Europe | LM | 2015 | Simvastatin 20mg | Som |  | 46.19 | LPG |  |  | 690.0 | 14.9 |
| Laos |  | WP | LM | 2013 | Simvastatin 20mg | Lao Kip |  | 20867 | LPG | 39000.0 | 1.9 | 25500.0 | 1.2 |
| Lebanon |  | EM | UM | 2013 | Atorvastatin 10mg | Lebanese Pounds |  | 22500 | LPG |  |  | 22038.0 | 1.0 |
|  |  |  |  | 2013 | Atorvastatin 10mg | Lebanese Pounds |  | 22500 | ORIG |  |  | 59093.0 | 2.6 |
|  |  |  |  | 2013 | Simvastatin 20mg | Lebanese Pounds |  | 22500 | ORIG |  |  | 40523.0 | 1.8 |
|  |  |  |  | 2013 | Simvastatin 20mg | Lebanese Pounds |  | 22500 | ORIG |  |  | 7428.2 | 0.3 |
| Mauritius |  | Africa | UM | 2008 | Simvastatin 20mg | Rupees |  | 215 | LPG |  |  | 223.5 | 1.0 |
|  |  |  |  | 2008 | Simvastatin 20mg | Rupees |  | 215 | ORIG |  |  | 414.2 | 1.9 |
| Mexico | Mexico City | Americas | UM | 2009 | Atorvastatin 10mg | Mexican Pesos |  | 57.64 | ORIG |  |  | 792.0 | 13.7 |
| Moldova |  | Europe | LM | 2011 | Simvastatin 20mg | Lei |  | 20 | LPG | 168.8 | 8.4 | 129.9 | 6.5 |
| Mongolia |  | WP | LM | 2012 | Atorvastatin 20mg | Tugrik |  | 6685.68 | LPG |  |  | 27000.0 | 4.0 |
|  |  |  |  | 2012 | Simvastatin 20mg | Tugrik |  | 6686 | LPG |  |  | 18000.0 | 2.7 |
| Nicaragua |  | Americas | LM | 2008 | Atorvastatin 10mg | Cordobas |  | 60.03 | LPG |  |  | 330.0 | 5.5 |
|  |  |  |  | 2008 | Simvastatin 20mg | Cordobas |  | 60.03 | LPG |  |  | 352.5 | 5.9 |
|  |  |  |  | 2008 | Atorvastatin 10mg | Cordobas |  | 60.03 | ORIG |  |  | 816.7 | 13.6 |
|  |  |  |  | 2008 | Simvastatin 20mg | Cordobas |  | 60.03 | ORIG |  |  | 945.0 | 15.7 |
| Oman |  | EM | High | 2007 | Simvastatin 20mg | Omani Rials |  | 3.5 | LPG |  |  | 11.7 | 3.3 |
|  |  |  |  | 2007 | Atorvastatin 20mg | Omani Rials |  | 3.5 | ORIG |  |  | 31.6 | 9.0 |
|  |  |  |  | 2007 | Simvastatin 20mg | Omani Rials |  | 3.5 | ORIG |  |  | 29.6 | 8.5 |
| Russia | Tatarstan | Europe | UM | 2011 | Atorvastatin 10mg | Roubles |  | 144 | LPG | 156.8 | 1.1 | 327.5 | 2.3 |
|  |  |  |  | 2011 | Simvastatin 20mg | Roubles |  | 144 | LPG | 137.0 | 1.0 | 359.5 | 2.5 |
|  |  |  |  | 2011 | Atorvastatin 10mg | Roubles |  | 144 | ORIG | 556.4 | 3.9 | 821.4 | 5.7 |
|  |  |  |  | 2011 | Simvastatin 20mg | Roubles |  | 144 | ORIG | 567.2 | 3.9 | 589.2 | 4.1 |
| Saudi Arabia |  | EM | High | 2015 | Atorvastatin 10mg | Saudi Riyals |  | 100 | LPG |  |  | 67.2 | 0.7 |
|  |  |  |  | 2015 | Lovastatin 10mg | Saudi Riyals |  | 100 | LPG |  |  | 53.8 | 0.5 |
|  |  |  |  | 2015 | Atorvastatin 10mg | Saudi Riyals |  | 100 | ORIG |  |  | 125.4 | 1.3 |
|  |  |  |  | 2015 | Lovastatin 10mg | Saudi Riyals |  | 100 | ORIG |  |  | 108.1 | 1.1 |
| Sudan |  | EM | LM | 2013 | Atorvastatin 20mg | Sudanese Pounds |  | 12 | LPG | 48.0 | 4.0 | 33.0 | 2.8 |
|  |  |  |  | 2012 | Atorvastatin 20mg | Sudanese Pounds |  | 12 | LPG | 28.2 | 2.4 | 23.5 | 2.0 |
|  |  |  |  | 2013 | Simvastatin 20mg | Sudanese Pounds |  | 12 | LPG | 25.2 | 2.1 | 24.0 | 2.0 |
|  |  |  |  | 2012 | Simvastatin 20mg | Sudanese Pounds |  | 12 | LPG |  |  | 19.5 | 1.6 |
| Tajikistan |  | Europe | Low | 2013 | Simvastatin 20mg | Somoni |  | 6.67 | LPG | 71.8 | 10.8 | 42.9 | 6.4 |
| Tanzania |  | Africa | Low | 2012 | Simvastatin 20mg | Tanzania Shillings |  | 5667 | LPG |  |  | 22500.0 | 4.0 |
| Ukraine |  | Europe | LM | 2012 | Atorvastatin 20mg | Hryvnia |  | 41.59 | LPG | 71.8 | 1.7 | 71.5 | 1.7 |
|  |  |  |  | 2012 | Simvastatin 20mg | Hryvnia |  | 41.59 | LPG | 60.0 | 1.4 | 57.6 | 1.4 |
|  |  |  |  | 2007 | Simvastatin 20mg | UAH |  | 14.67 | LPG | 51.3 | 3.5 | 102.0 | 7.0 |
|  |  |  |  | 2012 | Atorvastatin 20mg | Hryvnia |  | 41.59 | ORIG | 354.3 | 8.5 | 338.2 | 8.1 |
|  |  |  |  | 2012 | Simvastatin 20mg | Hryvnia |  | 41.59 | ORIG | 114.9 | 2.8 | 108.6 | 2.6 |
|  |  |  |  | 2007 | Simvastatin 20mg | UAH |  | 14.67 | ORIG |  |  | 127.5 | 8.7 |

Abbreviations used in the table: EM – Eastern Mediterranean, SEA – South-East Asia, WP – Western Pacific, LM – lower middle-income, UM – upper middle-income, LCU – local currency unit, LPG – lowest price generic, ORIG – originator.
