# Supplementary material for: Barriers to accessibility of medicines for hyperlipidemia in low- and middle-income countries
Source: PLOS Glob Public Health. 2024 Feb 12;4(2):e0002905. doi: 10.1371/journal.pgph.0002905 (PMC10861044; doi:10.1371/journal.pgph.0002905)
Supplement: S4 Table — (DOCX) [file pgph.0002905.s004.docx]

**S4 Table**: Price and affordability of statins from Health Action International (HAI) Database

| Country | Subregion | WHO Region | WB Income Classification | Year  of Survey | Drug and Dose | Local Currency Unit  (LCU) |  | Daily Wage, LCU | Originator or Lowest Price Generic | Public Sector | | Private Sector | |
| --- | --- | --- | --- | --- | --- | --- | --- | --- | --- | --- | --- | --- | --- |
|  |  |  |  |  |  |  |  |  |  | Median Treatment Price per month, LCU | No. of Days’ wage | Median Treatment Price per month, LCU | No. of Days’ wage |
| Afghanistan |  | EM | Low | 2011 | Atorvastatin 20mg | Afghani |  | 150 | LPG |  |  | 109.2 | 0.7 |
|  |  |  |  | 2011 | Simvastatin 20mg | Afghani |  | 150 | LPG | 120.0 | 0.8 | 160.0 | 1.1 |
| Brazil | Rio Grande do Sul | Americas | UM | 2008 | Simvastatin 20mg | Reals |  | 12.73 | LPG |  |  | 47.7 | 3.8 |
|  |  |  |  | 2008 | Atorvastatin 10mg | Reals |  | 12.73 | ORIG |  |  | 104.9 | 8.2 |
|  |  |  |  | 2008 | Simvastatin 20mg | Reals |  | 12.73 | ORIG |  |  | 106.4 | 8.4 |
| Burkina Faso |  | Africa | Low | 2009 | Simvastatin 20mg | FCFA |  | 1023 | LPG |  |  | 9920.0 | 9.7 |
|  |  |  |  | 2009 | Simvastatin 20mg | FCFA |  | 1023 | ORIG |  |  | 21412.5 | 20.9 |
| China | Shaanxi Province | WP | UM | 2012 | Atorvastatin 20mg | Yuan Renminbi |  | 33.33 | LPG | 223.5 | 6.7 | 53.5 | 1.6 |
|  | Shaanxi Province |  |  | 2014 | Atorvastatin 20mg | Yuan Renminbi |  | 37.33 | LPG | 203.5 | 5.5 | 192.8 | 5.2 |
|  | Shaanxi Province |  |  | 2012 | Simvastatin 20mg | Yuan Renminbi |  | 33.33 | LPG | 58.9 | 1.8 | 56.2 | 1.7 |
|  | Shaanxi Province |  |  | 2014 | Simvastatin 20mg | Yuan Renminbi |  | 37.33 | LPG | 32.2 | 0.9 | 47.1 | 1.3 |
|  | Shaanxi Province |  |  | 2010 | Simvastatin 20mg | Yuan Renminbi |  | 25.33 | LPG | 55.5 | 2.2 | 49.2 | 1.9 |
|  | Shaanxi Province |  |  | 2012 | Atorvastatin 20mg | Yuan Renminbi |  | 33.33 | ORIG | 315.4 | 9.5 | 255.0 | 7.7 |
|  | Shaanxi Province |  |  | 2014 | Atorvastatin 20mg | Yuan Renminbi |  | 37.33 | ORIG | 309.0 | 8.3 | 252.0 | 6.8 |
|  | Shaanxi Province |  |  | 2010 | Atorvastatin 20mg | Yuan Renminbi |  | 25.33 | ORIG | 315.4 | 12.5 | 278.5 | 11.0 |
|  | Shaanxi Province |  |  | 2012 | Simvastatin 20mg | Yuan Renminbi |  | 33.33 | ORIG | 107.1 | 3.2 | 102.8 | 3.1 |
|  | Shaanxi Province |  |  | 2014 | Simvastatin 20mg | Yuan Renminbi |  | 37.33 | ORIG | 103.6 | 2.8 | 98.1 | 2.6 |
|  | Shaanxi Province |  |  | 2010 | Simvastatin 20mg | Yuan Renminbi |  | 25.33 | ORIG | 138.0 | 5.4 | 113.5 | 4.5 |
| Colombia |  | Americas | UM | 2008 | Lovastatin 20mg | Colombian Pesos |  | 15383 | LPG |  |  | 9000.0 | 0.6 |
|  |  |  |  | 2008 | Simvastatin 20mg | Colombian Pesos |  | 15383 | LPG |  |  | 56100.0 | 3.6 |
|  |  |  |  | 2008 | Simvastatin 20mg | Colombian Pesos |  | 15383 | ORIG |  |  | 232800.0 | 15.1 |
| Ecuador |  | Americas | UM | 2008 | Atorvastatin 10mg | USD |  | 6.67 | LPG |  |  | 21.1 | 3.2 |
|  |  |  |  | 2008 | Simvastatin 20mg | USD |  | 6.67 | LPG |  |  | 20.7 | 3.1 |
|  |  |  |  | 2008 | Atorvastatin 10mg | USD |  | 6.67 | ORIG |  |  | 44.9 | 6.7 |
|  |  |  |  | 2008 | Simvastatin 20mg | USD |  | 6.67 | ORIG |  |  | 53.8 | 8.1 |
| Egypt |  | EM | LM | 2013 | Atorvastatin 10mg | Egyptian Pounds |  | 40 | LPG |  |  | 68.5 | 1.7 |
|  |  |  |  | 2013 | Simvastatin 20mg | Egyptian Pounds |  | 40 | LPG |  |  | 85.7 | 2.1 |
|  |  |  |  | 2013 | Atorvastatin 10mg | Egyptian Pounds |  | 40 | ORIG |  |  | 128.5 | 3.2 |
|  |  |  |  | 2013 | Simvastatin 20mg | Egyptian Pounds |  | 40 | ORIG |  |  | 171.4 | 4.3 |
| Haiti |  | Americas | Low | 2011 | Atorvastatin 10mg | Gourdes |  | 200 | LPG |  |  | 525.0 | 2.6 |
|  |  |  |  | 2011 | Simvastatin 20mg | Gourdes |  | 200 | LPG |  |  | 425.0 | 2.1 |
| India | NCT Delhi | SEA | LM | 2011 | Atorvastatin 10mg | Indian Rupees |  | 247 | LPG |  |  | 252.0 | 1.0 |
|  |  |  |  | 2011 | Simvastatin 20mg | Indian Rupees |  | 247 | LPG |  |  | 145.2 | 0.6 |
|  |  |  |  | 2011 | Atorvastatin 10mg | Indian Rupees |  | 247 | ORIG |  |  | 252.0 | 1.0 |
|  |  |  |  | 2011 | Simvastatin 20mg | Indian Rupees |  | 247 | ORIG |  |  | 540.0 | 2.2 |
| Indonesia |  | SEA | LM | 2010 | Simvastatin 20mg | Indonesian Rupiah |  | 36500 | LPG | 20253.9 | 6.0 | 21000.0 | 0.6 |
| Iran |  | EM | UM | 2007 | Atorvastatin 20mg | Iranian Rials |  | 108400 | LPG | 33000.0 | 0.3 | 33000.0 | 0.3 |
|  |  |  |  | 2007 | Simvastatin 20mg | Iranian Rials |  | 108400 | LPG | 30000.0 | 0.3 | 30000.0 | 0.3 |
|  |  |  |  | 2014 | Simvastatin 20mg | Iranian Rials |  | 270000 | LPG | 51000.0 | 0.2 | 51000.0 | 0.2 |
|  |  |  |  | 2007 | Atorvastatin 20mg | Iranian Rials |  | 108400 | ORIG |  |  | 912000.0 | 8.4 |
| Kyrgyzstan |  | Europe | LM | 2010 | Simvastatin 20mg | Som |  | 26.67 | LPG |  |  | 687.2 | 25.8 |
|  |  | Europe | LM | 2015 | Simvastatin 20mg | Som |  | 46.19 | LPG |  |  | 690.0 | 14.9 |
| Laos |  | WP | LM | 2013 | Simvastatin 20mg | Lao Kip |  | 20867 | LPG | 39000.0 | 1.9 | 25500.0 | 1.2 |
| Lebanon |  | EM | UM | 2013 | Atorvastatin 10mg | Lebanese Pounds |  | 22500 | LPG |  |  | 22038.0 | 1.0 |
|  |  |  |  | 2013 | Atorvastatin 10mg | Lebanese Pounds |  | 22500 | ORIG |  |  | 59093.0 | 2.6 |
|  |  |  |  | 2013 | Simvastatin 20mg | Lebanese Pounds |  | 22500 | ORIG |  |  | 40523.0 | 1.8 |
|  |  |  |  | 2013 | Simvastatin 20mg | Lebanese Pounds |  | 22500 | ORIG |  |  | 7428.2 | 0.3 |
| Mauritius |  | Africa | UM | 2008 | Simvastatin 20mg | Rupees |  | 215 | LPG |  |  | 223.5 | 1.0 |
|  |  |  |  | 2008 | Simvastatin 20mg | Rupees |  | 215 | ORIG |  |  | 414.2 | 1.9 |
| Mexico | Mexico City | Americas | UM | 2009 | Atorvastatin 10mg | Mexican Pesos |  | 57.64 | ORIG |  |  | 792.0 | 13.7 |
| Moldova |  | Europe | LM | 2011 | Simvastatin 20mg | Lei |  | 20 | LPG | 168.8 | 8.4 | 129.9 | 6.5 |
| Mongolia |  | WP | LM | 2012 | Atorvastatin 20mg | Tugrik |  | 6685.68 | LPG |  |  | 27000.0 | 4.0 |
|  |  |  |  | 2012 | Simvastatin 20mg | Tugrik |  | 6686 | LPG |  |  | 18000.0 | 2.7 |
| Nicaragua |  | Americas | LM | 2008 | Atorvastatin 10mg | Cordobas |  | 60.03 | LPG |  |  | 330.0 | 5.5 |
|  |  |  |  | 2008 | Simvastatin 20mg | Cordobas |  | 60.03 | LPG |  |  | 352.5 | 5.9 |
|  |  |  |  | 2008 | Atorvastatin 10mg | Cordobas |  | 60.03 | ORIG |  |  | 816.7 | 13.6 |
|  |  |  |  | 2008 | Simvastatin 20mg | Cordobas |  | 60.03 | ORIG |  |  | 945.0 | 15.7 |
| Oman |  | EM | High | 2007 | Simvastatin 20mg | Omani Rials |  | 3.5 | LPG |  |  | 11.7 | 3.3 |
|  |  |  |  | 2007 | Atorvastatin 20mg | Omani Rials |  | 3.5 | ORIG |  |  | 31.6 | 9.0 |
|  |  |  |  | 2007 | Simvastatin 20mg | Omani Rials |  | 3.5 | ORIG |  |  | 29.6 | 8.5 |
| Russia | Tatarstan | Europe | UM | 2011 | Atorvastatin 10mg | Roubles |  | 144 | LPG | 156.8 | 1.1 | 327.5 | 2.3 |
|  |  |  |  | 2011 | Simvastatin 20mg | Roubles |  | 144 | LPG | 137.0 | 1.0 | 359.5 | 2.5 |
|  |  |  |  | 2011 | Atorvastatin 10mg | Roubles |  | 144 | ORIG | 556.4 | 3.9 | 821.4 | 5.7 |
|  |  |  |  | 2011 | Simvastatin 20mg | Roubles |  | 144 | ORIG | 567.2 | 3.9 | 589.2 | 4.1 |
| Saudi Arabia |  | EM | High | 2015 | Atorvastatin 10mg | Saudi Riyals |  | 100 | LPG |  |  | 67.2 | 0.7 |
|  |  |  |  | 2015 | Lovastatin 10mg | Saudi Riyals |  | 100 | LPG |  |  | 53.8 | 0.5 |
|  |  |  |  | 2015 | Atorvastatin 10mg | Saudi Riyals |  | 100 | ORIG |  |  | 125.4 | 1.3 |
|  |  |  |  | 2015 | Lovastatin 10mg | Saudi Riyals |  | 100 | ORIG |  |  | 108.1 | 1.1 |
| Sudan |  | EM | LM | 2013 | Atorvastatin 20mg | Sudanese Pounds |  | 12 | LPG | 48.0 | 4.0 | 33.0 | 2.8 |
|  |  |  |  | 2012 | Atorvastatin 20mg | Sudanese Pounds |  | 12 | LPG | 28.2 | 2.4 | 23.5 | 2.0 |
|  |  |  |  | 2013 | Simvastatin 20mg | Sudanese Pounds |  | 12 | LPG | 25.2 | 2.1 | 24.0 | 2.0 |
|  |  |  |  | 2012 | Simvastatin 20mg | Sudanese Pounds |  | 12 | LPG |  |  | 19.5 | 1.6 |
| Tajikistan |  | Europe | Low | 2013 | Simvastatin 20mg | Somoni |  | 6.67 | LPG | 71.8 | 10.8 | 42.9 | 6.4 |
| Tanzania |  | Africa | Low | 2012 | Simvastatin 20mg | Tanzania Shillings |  | 5667 | LPG |  |  | 22500.0 | 4.0 |
| Ukraine |  | Europe | LM | 2012 | Atorvastatin 20mg | Hryvnia |  | 41.59 | LPG | 71.8 | 1.7 | 71.5 | 1.7 |
|  |  |  |  | 2012 | Simvastatin 20mg | Hryvnia |  | 41.59 | LPG | 60.0 | 1.4 | 57.6 | 1.4 |
|  |  |  |  | 2007 | Simvastatin 20mg | UAH |  | 14.67 | LPG | 51.3 | 3.5 | 102.0 | 7.0 |
|  |  |  |  | 2012 | Atorvastatin 20mg | Hryvnia |  | 41.59 | ORIG | 354.3 | 8.5 | 338.2 | 8.1 |
|  |  |  |  | 2012 | Simvastatin 20mg | Hryvnia |  | 41.59 | ORIG | 114.9 | 2.8 | 108.6 | 2.6 |
|  |  |  |  | 2007 | Simvastatin 20mg | UAH |  | 14.67 | ORIG |  |  | 127.5 | 8.7 |

Abbreviations used in the table: EM – Eastern Mediterranean, SEA – South-East Asia, WP – Western Pacific, LM – lower middle-income, UM – upper middle-income, LCU – local currency unit, LPG – lowest price generic, ORIG – originator.
